# (1*H*-Benzimidazole-5-carb­oxy­lic acid-κ*N* ^3^)(1*H*-benzimidazole-6-carb­oxy­lic acid-κ*N* ^3^)silver(I) perchlorate

**DOI:** 10.1107/S1600536811010427

**Published:** 2011-03-26

**Authors:** Li Ma, Yu-Hua Huang, Jian-Feng Xu, Hong Deng

**Affiliations:** a School of Chemistry and Environment, South China Nomal University, Guangzhou 510006, People’s Republic of China

## Abstract

The reaction of 1*H*-benzimidazole-5-carb­oxy­lic acid with silver nitrate in the presence of perchloric acid under hydro­thermal conditions yielded the title complex, [Ag(C_8_H_6_N_2_O_2_)_2_]ClO_4_, which comprises of an [Ag(C_8_H_6_N_2_O_2_)_2_] mononuclear cation and a perchlorate anion. The Ag^I^ ion is coordinated by two N atoms from two different neutral 1*H*-benzimidazole-5-carb­oxy­lic acid ligands with an N—Ag—N bond angle of 163.21 (14)°, forming an [Ag(C_8_H_6_N_2_O_2_)_2_] mononuclear cation. Although both ligands in the mononuclear cation are monodentate with one N atom coordinated to the metal ion, they are different: one is N^3^ coordinated to the Ag ^I^ ion and the N^1^ atom protonated, the other with the N^1^ coordinated to the Ag ^I^ ion and the N^3^ atom protonated (and thus formally a 1*H*-benzimidazole-6-carb­oxy­lic acid rather than a 1*H*-benzimidazole-5-carb­oxy­lic acid ligand). The planes of the two planar ligands are roughly perpendicular, making a dihedral angle of 84.97 (2)°. The packing of the ions is stablized by extensive O—H⋯O, N—H⋯O and C—H⋯O hydrogen bonds, and by remote Ag⋯O inter­actions [3.002 (3), 3.581 (5) and 3.674 (5) Å].

## Related literature

For related structures, see: Guo, Cao *et al.* (2007 ▶); Guo, Li *et al.* (2007 ▶); Liu *et al.* (2005 ▶); Peng, Ma *et al.* (2010 ▶); Peng, Qiu *et al.* (2010 ▶). For graph-set motifs of hydrogen bonds, see: Bernstein *et al.* (1995 ▶); Eppel & Bernstein (2008 ▶); Grell *et al.* (1999 ▶). For van der Waals radii, see: Bondi (1964 ▶).
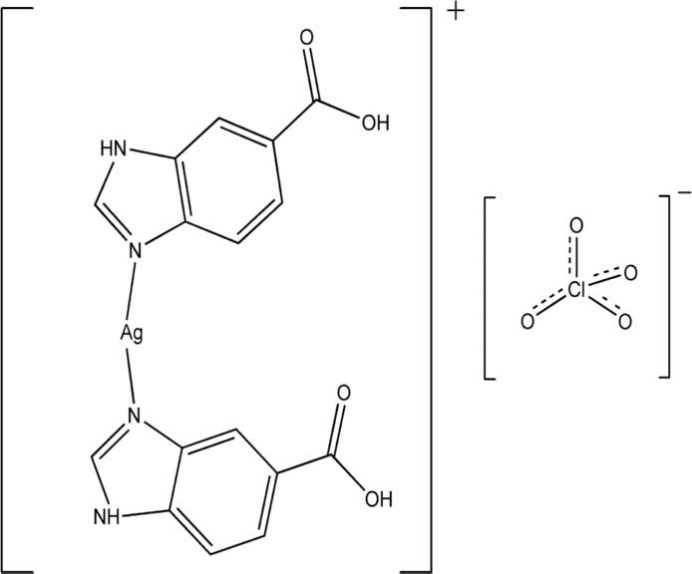


## Experimental

### 

#### Crystal data

[Ag(C_8_H_6_N_2_O_2_)_2_]ClO_4_
*M* *_r_* = 531.62
Triclinic, 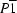

*a* = 4.933 (2) Å
*b* = 13.330 (5) Å
*c* = 14.498 (6) Å
α = 78.566 (5)°
β = 89.111 (5)°
γ = 82.554 (5)°
*V* = 926.5 (6) Å^3^
*Z* = 2
Mo *K*α radiation
μ = 1.29 mm^−1^
*T* = 296 K
0.26 × 0.24 × 0.22 mm

#### Data collection

Bruker SMART APEX CCD diffractometer
Absorption correction: multi-scan (*SADABS*; Sheldrick, 1996 ▶) *T*_min_ = 0.746, *T*_max_ = 0.774
4604 measured reflections
3247 independent reflections
2630 reflections with *I* > 2σ(*I*)
*R*_int_ = 0.017

#### Refinement

*R*[*F* ^2^ > 2σ(*F* ^2^)] = 0.040
*wR*(*F* ^2^) = 0.103
*S* = 1.04
3247 reflections
273 parameters
H-atom parameters constrained
Δρ_max_ = 0.62 e Å^−3^
Δρ_min_ = −0.70 e Å^−3^

### 

Data collection: *APEX2* (Bruker, 2004 ▶); cell refinement: *SAINT* (Bruker, 2004 ▶); data reduction: *SAINT*; program(s) used to solve structure: *SHELXS97* (Sheldrick, 2008 ▶); program(s) used to refine structure: *SHELXL97* (Sheldrick, 2008 ▶); molecular graphics: *SHELXTL* (Sheldrick, 2008 ▶); software used to prepare material for publication: *SHELXTL*.

## Supplementary Material

Crystal structure: contains datablocks I, global. DOI: 10.1107/S1600536811010427/zl2355sup1.cif

Structure factors: contains datablocks I. DOI: 10.1107/S1600536811010427/zl2355Isup2.hkl

Additional supplementary materials:  crystallographic information; 3D view; checkCIF report

## Figures and Tables

**Table 1:** Hydrogen-bond geometry (Å, °)

*D*—H⋯*A*	*D*—H	H⋯*A*	*D*⋯*A*	*D*—H⋯*A*
O1—H1*A*⋯O2^i^	0.82	1.82	2.634 (5)	173
N2—H2⋯O5^ii^	0.86	2.15	2.983 (6)	163
O3—H3⋯O4^iii^	0.82	1.80	2.608 (6)	168
N4—H4*A*⋯O6^iv^	0.86	2.14	2.935 (6)	153
C14—H14⋯O1^v^	0.93	2.60	3.491 (6)	162
C15—H15⋯O2^vi^	0.93	2.51	3.398 (5)	161

Symmetry codes: (i) 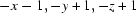; (ii) 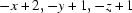; (iii) 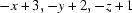; (iv) 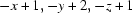; (v) 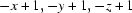; (vi) 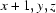.

**Fig. 1.**
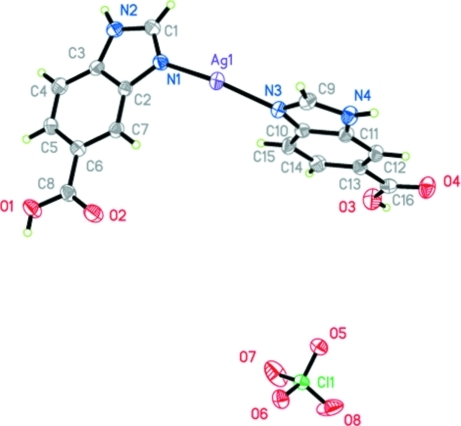
The structure of the title compound, showing the atomic numbering scheme. Non-H atoms are shown with 30% probability displacement ellipsoids.

**Fig. 2.**
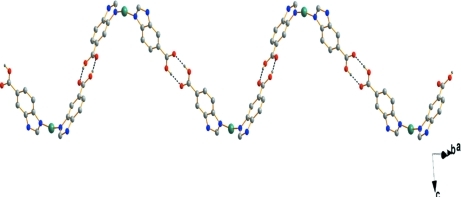
One of the ribbon like [Ag(C8H6N2O2)2] chains linked by O—H···O hydrogen bonds along the (1 1 0) direction.

**Fig. 3.**
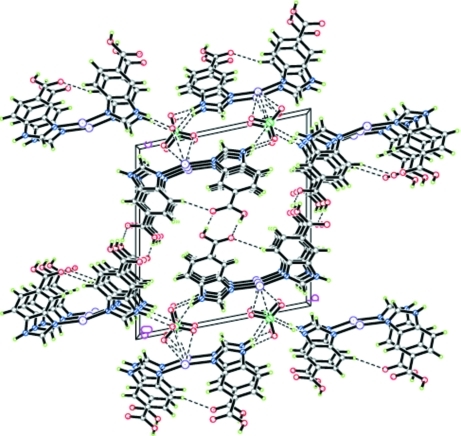
A packing view of title compound along the a axis, showing the O—H···O, O—H···N and C—H···O hydrogen bonds and the Ag···O interactions.

## supplementary crystallographic information

### Comment

In recent years, N-heterocyclic carboxylic acids as organic ligands attracted significant attention not only due to their versatile coordination modes but also because of their ability to facilitate the formation of high-dimensional coordination compounds. 1*H*-benzimidazole-5-carboxylic acid is such an organic ligand, with four donor atoms (two N and two O atoms) and variable coordination modes. As a potential multidentate ligand, 1*H*-benzimidazole-5-carboxylic acid is an excellent candidate for the construction of three-dimensional architectures. Up to this date, reported compounds based on this ligand are still quite rare, but have recently started to attract some interest (Guo, Cao *et al.* (2007); Guo, Li *et al.* (2007); Liu *et al.* (2005); Peng, Ma *et al.* (2010); Peng, Qiu *et al.* (2010).). According to these precedents in the literature and experience, the nature of the final metal coordination framework is still hard to predict. To gain more insight into this system we thus chose 1*H*-benzimidazole-5-carboxylic acid and silver nitrate in a 1:1 ratio in the presence of perchloric acid under hydrothermal conditions to construct a new framework. Herein, we report an Ag coordination complex salt based on this ligand, namely [Ag(C_8_H_6_N_2_O_2_)_2_].ClO_4_, with a three-dimensional supramolecular network created by O—H···O, N—H···O, and C—H···O hydrogen bonds, and by Ag···O interactions [3.002 (3), 3.581 (5) and 3.674 (5) Å].

As shown in Figure 1, the title compound contains an Ag^I^ ion, two silver coordinated different neutral 1*H*-benzimidazole-5-carboxylic acid ligands and a perchlorate anion. The Ag^I^ ion is coordinated by two N atoms from two different 1*H*-benzimidazole-5-carboxylic acid ligands with Ag—N bond lengths of 2.106 (4) Å, and an N—Ag—N bond angle 163.23 °. Although both of the two ligands in the mononuclear cation are monodentate ligands with one nitrogen atom coordinated to the metal ion, they are different: one with the N^3^ coordinated to the Ag ^I^ ion and the N^1^ atom protonated, the other with the N^1^ coordinated to the Ag ^I^ ion and the N^3^ atom protonated (and thus formally a 1*H*-benzimidazole -6-carboxylic acid rather than a 1*H*-benzimidazole-5-carboxylic acid ligand), thus forming a mononuclear [Ag(C_8_H_6_N_2_O_2_)_2_] cation.

Adjacent [Ag(C_8_H_6_N_2_O_2_)_2_] mononuclear cations are linked to each other through O—H···O hydrogen bonds with the *R*^2^_2_8 graph set motif typical for carboxylic acids (Bernstein *et al.* (1995); Eppel & Bernstein (2008); Grell *et al.* (1999)) to form a ribbon-like chain of [Ag(C_8_H_6_N_2_O_2_)_2_] units that stretch along the (1 1 0) direction of the structure (Figure 2). Adjacent chains are further linked to each other by weak C—H···O hydrogen bonds leading to the formation of layers of [Ag(C_8_H_6_N_2_O_2_)_2_] cations. The perchlorate anions are bonded to these layers through strong N—H···O hydrogen bonds originating from the imidazole rings, and through short O···Ag interactions (Figure 3). The O6···Ag, O7···Ag, O8···Ag, distances are 3.002 (3), 3.581 (5), 3.674 (5) Å, respectively, which is shorter than the sum of the van der Waals radii for silver and oxygen atoms (3.91 Å) (Bondi (1964)).

### Experimental

An aqueous mixture (10 ml) of 1*H*-benzimidazole-5-carboxylic acid (0.05 g 0.3 mmol), silver nitrate (0.05 g 0.3 mmol) and perchloric acid (pH=2) was placed in a 23 ml Teflon-lined stainless-steel autoclave, heated to 443 K for 3 days, then cooled to room temperature at 5 K/h. Yellow prism-shaped single crystals were collected (yield 0.06 g, 0.1 mmol, 75% based on 1*H*-benzimidazole-5-carboxylic acid).

### Refinement

All H-atoms attached to C, N and O atoms were fixed geometrically and treated as riding with C—H = 0.93 Å, N—H = 0.86 Å and O—H = 0.82 Å and *U*_iso_(H) = 1.2*U*_eq_(C,*N*) and *U*_iso_(H) = 1.5*U*_eq_(O).

### Crystal data

**Table:**

[Ag(C_8_H_6_N_2_O_2_)_2_]ClO_4_	*Z* = 2
*M**_r_* = 531.62	*F*(000) = 528.0
Triclinic, *P*1	*D*_x_ = 1.906 Mg m^−^^3^
Hall symbol: -P 1	Mo *K*α radiation, λ = 0.71073 Å
*a* = 4.933 (2) Å	Cell parameters from 3317 reflections
*b* = 13.330 (5) Å	θ = 1.6–25.2°
*c* = 14.498 (6) Å	µ = 1.29 mm^−^^1^
α = 78.566 (5)°	*T* = 296 K
β = 89.111 (5)°	Prism, yellow
γ = 82.554 (5)°	0.26 × 0.24 × 0.22 mm
*V* = 926.5 (6) Å^3^	

### Data collection

**Table:**

Bruker SMART APEX CCD diffractometer	3247 independent reflections
Radiation source: fine-focus sealed tube	2630 reflections with *I* > 2σ(*I*)
graphite	*R*_int_ = 0.017
ω scans	θ_max_ = 25.2°, θ_min_ = 1.6°
Absorption correction: multi-scan (*SADABS*; Sheldrick, 1996)	*h* = −5→5
*T*_min_ = 0.746, *T*_max_ = 0.774	*k* = −15→13
4604 measured reflections	*l* = −16→17

### Refinement

**Table:**

Refinement on *F*^2^	Primary atom site location: structure-invariant direct methods
Least-squares matrix: full	Secondary atom site location: difference Fourier map
*R*[*F*^2^ > 2σ(*F*^2^)] = 0.040	Hydrogen site location: inferred from neighbouring sites
*wR*(*F*^2^) = 0.103	H-atom parameters constrained
*S* = 1.04	*w* = 1/[σ^2^(*F*_o_^2^) + (0.0581*P*)^2^ + 0.3563*P*] where *P* = (*F*_o_^2^ + 2*F*_c_^2^)/3
3247 reflections	(Δ/σ)_max_ = 0.001
273 parameters	Δρ_max_ = 0.62 e Å^−^^3^
0 restraints	Δρ_min_ = −0.70 e Å^−^^3^

### Special details

**Table:**

Geometry. All e.s.d.'s (except the e.s.d. in the dihedral angle between two l.s. planes) are estimated using the full covariance matrix. The cell e.s.d.'s are taken into account individually in the estimation of e.s.d.'s in distances, angles and torsion angles; correlations between e.s.d.'s in cell parameters are only used when they are defined by crystal symmetry. An approximate (isotropic) treatment of cell e.s.d.'s is used for estimating e.s.d.'s involving l.s. planes.
Refinement. Refinement of *F*^2^ against ALL reflections. The weighted *R*-factor *wR* and goodness of fit *S* are based on *F*^2^, conventional *R*-factors *R* are based on *F*, with *F* set to zero for negative *F*^2^. The threshold expression of *F*^2^ > σ(*F*^2^) is used only for calculating *R*-factors(gt) *etc*. and is not relevant to the choice of reflections for refinement. *R*-factors based on *F*^2^ are statistically about twice as large as those based on *F*, and *R*- factors based on ALL data will be even larger.

### Fractional atomic coordinates and isotropic or equivalent isotropic displacement parameters (Å^2^)

**Table:**

	*x*	*y*	*z*	*U*_iso_*/*U*_eq_	
Ag1	0.44123 (8)	0.70811 (3)	0.15898 (2)	0.04721 (15)	
C1	0.6189 (10)	0.4842 (4)	0.1256 (3)	0.0466 (11)	
H1	0.7430	0.5057	0.0793	0.056*	
C2	0.3124 (8)	0.4843 (3)	0.2325 (3)	0.0346 (9)	
C3	0.3830 (9)	0.3825 (3)	0.2238 (3)	0.0408 (10)	
C4	0.2654 (10)	0.3021 (4)	0.2792 (3)	0.0509 (12)	
H4	0.3152	0.2338	0.2738	0.061*	
C5	0.0729 (10)	0.3286 (3)	0.3421 (3)	0.0471 (11)	
H5	−0.0092	0.2766	0.3804	0.057*	
C6	−0.0052 (9)	0.4309 (3)	0.3509 (3)	0.0396 (10)	
C7	0.1163 (9)	0.5103 (3)	0.2959 (3)	0.0391 (10)	
H7	0.0675	0.5786	0.3016	0.047*	
C8	−0.2186 (10)	0.4595 (3)	0.4175 (3)	0.0446 (11)	
C9	0.4393 (10)	0.9464 (4)	0.1285 (3)	0.0456 (11)	
H9	0.3170	0.9604	0.0780	0.055*	
C10	0.6828 (9)	0.8675 (3)	0.2492 (3)	0.0371 (10)	
C11	0.7374 (9)	0.9692 (3)	0.2309 (3)	0.0381 (10)	
C12	0.9185 (9)	1.0038 (3)	0.2866 (3)	0.0421 (10)	
H12	0.9543	1.0719	0.2747	0.050*	
C13	1.0426 (9)	0.9313 (3)	0.3608 (3)	0.0390 (10)	
C14	0.9860 (10)	0.8291 (4)	0.3798 (3)	0.0482 (11)	
H14	1.0709	0.7827	0.4308	0.058*	
C15	0.8066 (10)	0.7964 (3)	0.3241 (3)	0.0460 (11)	
H15	0.7694	0.7284	0.3364	0.055*	
C16	1.2386 (10)	0.9636 (4)	0.4219 (3)	0.0457 (11)	
Cl1	0.9204 (2)	0.77011 (9)	0.97296 (8)	0.0448 (3)	
N1	0.4642 (7)	0.5474 (3)	0.1693 (2)	0.0408 (9)	
N2	0.5791 (8)	0.3854 (3)	0.1548 (3)	0.0493 (10)	
H2	0.6610	0.3333	0.1343	0.059*	
N3	0.4956 (8)	0.8550 (3)	0.1829 (2)	0.0415 (9)	
N4	0.5779 (8)	1.0176 (3)	0.1536 (3)	0.0470 (9)	
H4A	0.5686	1.0815	0.1264	0.056*	
O1	−0.3280 (8)	0.3835 (2)	0.4671 (3)	0.0581 (9)	
H1A	−0.4557	0.4062	0.4972	0.087*	
O2	−0.2846 (7)	0.5503 (2)	0.4241 (2)	0.0608 (10)	
O3	1.3333 (8)	0.8956 (3)	0.4925 (3)	0.0672 (10)	
H3	1.4522	0.9178	0.5187	0.101*	
O4	1.3026 (8)	1.0534 (3)	0.4028 (2)	0.0630 (10)	
O5	1.0476 (7)	0.7959 (3)	0.8839 (2)	0.0603 (9)	
O6	0.6290 (6)	0.7811 (3)	0.9620 (2)	0.0577 (9)	
O7	1.0171 (10)	0.6657 (4)	1.0117 (4)	0.1079 (18)	
O8	0.9910 (9)	0.8361 (5)	1.0309 (4)	0.117 (2)	

### Atomic displacement parameters (Å^2^)

**Table:**

	*U*^11^	*U*^22^	*U*^33^	*U*^12^	*U*^13^	*U*^23^
Ag1	0.0531 (3)	0.0393 (2)	0.0526 (2)	−0.01424 (16)	0.00379 (16)	−0.01219 (16)
C1	0.042 (3)	0.052 (3)	0.049 (3)	−0.009 (2)	0.008 (2)	−0.013 (2)
C2	0.031 (2)	0.035 (2)	0.038 (2)	−0.0045 (17)	−0.0032 (17)	−0.0080 (18)
C3	0.043 (3)	0.039 (2)	0.043 (2)	−0.005 (2)	0.0001 (19)	−0.014 (2)
C4	0.060 (3)	0.033 (2)	0.060 (3)	−0.004 (2)	0.006 (2)	−0.013 (2)
C5	0.056 (3)	0.035 (2)	0.050 (3)	−0.013 (2)	0.006 (2)	−0.005 (2)
C6	0.039 (3)	0.038 (2)	0.040 (2)	−0.0033 (19)	−0.0002 (19)	−0.0044 (19)
C7	0.041 (3)	0.031 (2)	0.046 (2)	−0.0022 (19)	0.0006 (19)	−0.0109 (19)
C8	0.047 (3)	0.038 (3)	0.047 (3)	−0.008 (2)	0.005 (2)	−0.002 (2)
C9	0.048 (3)	0.048 (3)	0.042 (2)	−0.006 (2)	−0.003 (2)	−0.013 (2)
C10	0.038 (3)	0.037 (2)	0.037 (2)	−0.0034 (19)	0.0048 (18)	−0.0102 (18)
C11	0.041 (3)	0.035 (2)	0.038 (2)	−0.0028 (19)	−0.0024 (19)	−0.0081 (18)
C12	0.047 (3)	0.034 (2)	0.045 (3)	−0.005 (2)	−0.003 (2)	−0.007 (2)
C13	0.035 (2)	0.041 (2)	0.040 (2)	−0.0063 (19)	0.0032 (18)	−0.0078 (19)
C14	0.057 (3)	0.043 (3)	0.040 (3)	−0.001 (2)	−0.004 (2)	−0.001 (2)
C15	0.057 (3)	0.032 (2)	0.049 (3)	−0.008 (2)	−0.001 (2)	−0.006 (2)
C16	0.048 (3)	0.051 (3)	0.038 (2)	−0.002 (2)	0.000 (2)	−0.010 (2)
Cl1	0.0334 (6)	0.0542 (7)	0.0455 (6)	−0.0021 (5)	−0.0026 (5)	−0.0084 (5)
N1	0.041 (2)	0.038 (2)	0.044 (2)	−0.0047 (17)	0.0058 (16)	−0.0087 (17)
N2	0.055 (3)	0.040 (2)	0.056 (2)	−0.0027 (18)	0.0131 (19)	−0.0196 (18)
N3	0.046 (2)	0.037 (2)	0.043 (2)	−0.0096 (17)	0.0016 (16)	−0.0109 (17)
N4	0.058 (3)	0.033 (2)	0.047 (2)	−0.0052 (18)	−0.0090 (18)	−0.0014 (17)
O1	0.059 (2)	0.0433 (19)	0.068 (2)	−0.0067 (16)	0.0241 (17)	−0.0017 (16)
O2	0.071 (3)	0.0371 (19)	0.071 (2)	−0.0037 (17)	0.0301 (19)	−0.0084 (16)
O3	0.079 (3)	0.066 (2)	0.054 (2)	−0.016 (2)	−0.0255 (19)	−0.0008 (19)
O4	0.072 (3)	0.054 (2)	0.064 (2)	−0.0176 (19)	−0.0196 (18)	−0.0074 (18)
O5	0.060 (2)	0.067 (2)	0.052 (2)	−0.0115 (19)	0.0080 (16)	−0.0069 (17)
O6	0.0317 (18)	0.064 (2)	0.075 (2)	−0.0046 (16)	−0.0046 (15)	−0.0080 (18)
O7	0.077 (3)	0.087 (3)	0.125 (4)	0.011 (3)	0.004 (3)	0.051 (3)
O8	0.060 (3)	0.199 (6)	0.128 (4)	−0.023 (3)	0.010 (3)	−0.120 (4)

### Geometric parameters (Å, °)

**Table:**

Ag1—N1	2.106 (4)	C10—C15	1.383 (6)
Ag1—N3	2.106 (4)	C10—C11	1.390 (6)
C1—N1	1.313 (6)	C10—N3	1.391 (5)
C1—N2	1.340 (6)	C11—N4	1.379 (5)
C1—H1	0.9300	C11—C12	1.393 (6)
C2—C7	1.383 (6)	C12—C13	1.386 (6)
C2—C3	1.387 (6)	C12—H12	0.9300
C2—N1	1.394 (5)	C13—C14	1.399 (6)
C3—N2	1.379 (6)	C13—C16	1.480 (6)
C3—C4	1.392 (6)	C14—C15	1.373 (6)
C4—C5	1.368 (7)	C14—H14	0.9300
C4—H4	0.9300	C15—H15	0.9300
C5—C6	1.399 (6)	C16—O4	1.255 (6)
C5—H5	0.9300	C16—O3	1.275 (5)
C6—C7	1.390 (6)	Cl1—O8	1.408 (4)
C6—C8	1.482 (6)	Cl1—O7	1.416 (4)
C7—H7	0.9300	Cl1—O5	1.426 (4)
C8—O2	1.234 (5)	Cl1—O6	1.434 (3)
C8—O1	1.294 (5)	N2—H2	0.8600
C9—N3	1.312 (6)	N4—H4A	0.8600
C9—N4	1.348 (6)	O1—H1A	0.8200
C9—H9	0.9300	O3—H3	0.8200
			
N1—Ag1—N3	163.21 (14)	C13—C12—H12	121.7
N1—C1—N2	112.9 (4)	C11—C12—H12	121.7
N1—C1—H1	123.6	C12—C13—C14	121.9 (4)
N2—C1—H1	123.6	C12—C13—C16	118.8 (4)
C7—C2—C3	121.0 (4)	C14—C13—C16	119.3 (4)
C7—C2—N1	129.7 (4)	C15—C14—C13	120.8 (4)
C3—C2—N1	109.3 (4)	C15—C14—H14	119.6
N2—C3—C2	105.3 (4)	C13—C14—H14	119.6
N2—C3—C4	132.8 (4)	C14—C15—C10	118.1 (4)
C2—C3—C4	122.0 (4)	C14—C15—H15	121.0
C5—C4—C3	116.6 (4)	C10—C15—H15	121.0
C5—C4—H4	121.7	O4—C16—O3	123.8 (4)
C3—C4—H4	121.7	O4—C16—C13	120.1 (4)
C4—C5—C6	122.4 (4)	O3—C16—C13	116.1 (4)
C4—C5—H5	118.8	O8—Cl1—O7	111.1 (4)
C6—C5—H5	118.8	O8—Cl1—O5	108.7 (3)
C7—C6—C5	120.4 (4)	O7—Cl1—O5	107.5 (3)
C7—C6—C8	117.4 (4)	O8—Cl1—O6	109.9 (2)
C5—C6—C8	122.3 (4)	O7—Cl1—O6	109.1 (3)
C2—C7—C6	117.7 (4)	O5—Cl1—O6	110.6 (2)
C2—C7—H7	121.2	C1—N1—C2	105.0 (4)
C6—C7—H7	121.2	C1—N1—Ag1	131.1 (3)
O2—C8—O1	123.2 (4)	C2—N1—Ag1	123.9 (3)
O2—C8—C6	121.2 (4)	C1—N2—C3	107.5 (4)
O1—C8—C6	115.6 (4)	C1—N2—H2	126.2
N3—C9—N4	112.7 (4)	C3—N2—H2	126.2
N3—C9—H9	123.7	C9—N3—C10	105.3 (4)
N4—C9—H9	123.7	C9—N3—Ag1	130.1 (3)
C15—C10—C11	121.0 (4)	C10—N3—Ag1	121.7 (3)
C15—C10—N3	129.7 (4)	C9—N4—C11	107.4 (4)
C11—C10—N3	109.2 (4)	C9—N4—H4A	126.3
N4—C11—C10	105.4 (4)	C11—N4—H4A	126.3
N4—C11—C12	133.0 (4)	C8—O1—H1A	109.5
C10—C11—C12	121.7 (4)	C16—O3—H3	109.5
C13—C12—C11	116.5 (4)		

### Hydrogen-bond geometry (Å, °)

**Table:**

*D*—H···*A*	*D*—H	H···*A*	*D*···*A*	*D*—H···*A*
O1—H1A···O2^i^	0.82	1.82	2.634 (5)	173
N2—H2···O5^ii^	0.86	2.15	2.983 (6)	163
O3—H3···O4^iii^	0.82	1.80	2.608 (6)	168
N4—H4A···O6^iv^	0.86	2.14	2.935 (6)	153
C14—H14···O3	0.93	2.41	2.729 (6)	100
C14—H14···O1^v^	0.93	2.60	3.491 (6)	162
C15—H15···O2^vi^	0.93	2.51	3.398 (5)	161

Symmetry codes: (i) −*x*−1, −*y*+1, −*z*+1; (ii) −*x*+2, −*y*+1, −*z*+1; (iii) −*x*+3, −*y*+2, −*z*+1; (iv) −*x*+1, −*y*+2, −*z*+1; (v) −*x*+1, −*y*+1, −*z*+1; (vi) *x*+1, *y*, *z*.
